# Spatial Distribution of Tree Species Governs the Spatio-Temporal Interaction of Leaf Area Index and Soil Moisture across a Forested Landscape

**DOI:** 10.1371/journal.pone.0058704

**Published:** 2013-03-12

**Authors:** Kusum J. Naithani, Doug C. Baldwin, Katie P. Gaines, Henry Lin, David M. Eissenstat

**Affiliations:** 1 Department of Geography, The Pennsylvania State University, University Park, Pennsylvania, United States of America; 2 Intercollege Graduate Degree Program in Ecology, The Pennsylvania State University, University Park, Pennsylvania, United States of America; 3 Department of Ecosystem Science and Management, The Pennsylvania State University, University Park, Pennsylvania, United States of America; Wuhan Botanical Garden, Chinese Academy of Sciences, China

## Abstract

Quantifying coupled spatio-temporal dynamics of phenology and hydrology and understanding underlying processes is a fundamental challenge in ecohydrology. While variation in phenology and factors influencing it have attracted the attention of ecologists for a long time, the influence of biodiversity on coupled dynamics of phenology and hydrology across a landscape is largely untested. We measured leaf area index (*L*) and volumetric soil water content (*θ*) on a co-located spatial grid to characterize forest phenology and hydrology across a forested catchment in central Pennsylvania during 2010. We used hierarchical Bayesian modeling to quantify spatio-temporal patterns of *L* and *θ*. Our results suggest that the spatial distribution of tree species across the landscape created unique spatio-temporal patterns of *L*, which created patterns of water demand reflected in variable soil moisture across space and time. We found a lag of about 11 days between increase in *L* and decline in *θ*. Vegetation and soil moisture become increasingly homogenized and coupled from leaf-onset to maturity but heterogeneous and uncoupled from leaf maturity to senescence. Our results provide insight into spatio-temporal coupling between biodiversity and soil hydrology that is useful to enhance ecohydrological modeling in humid temperate forests.

## Introduction

Vegetation plays an important role in movement of water across the landscape by exchanging water between the soil and the atmosphere via change in surface albedo and roughness [1], canopy water interception [2], and transpiration [3]–[5]; changing the hydro-mechanical properties of soil [6], [7]; and redistributing water laterally and vertically in soil profile via hydraulic redistribution [8]–[10]. On the other hand, survival and distribution of plants on a landscape depend on spatio-temporal patterns of soil water availability [11]–[14]. Therefore, an increased understanding of spatio-temporal patterns of vegetation water use and underlying mechanisms is critical for effective watershed management and advancement of the field of ecohydrology [15], [16]. Recent studies from arid ecosystems have reported the strong influence of spatio-temporal patterns of vegetation on horizontal and vertical gradients of soil moisture [17]–[19]. However, the underlying processes that create spatial and temporal patterns of leaf area index and soil moisture remain poorly understood, especially in humid regions. Understanding the governing factors of this interaction is critical for modeling carbon, water, and energy cycles at the landscape scale.

Leaf surface is the site of gaseous (water and CO_2_) exchange, therefore leaf area controls terrestrial water, energy and CO_2_ fluxes [4], [20]. Leaf area index (*L*), defined as half of the total intercepting leaf area (m^2^) per unit ground surface area (m^2^) [21], is used as a key input to a variety of ecosystem and hydrologic models [22] to incorporate phenological changes. Similarly, volumetric soil water content (*θ*) is a commonly used input in hydrologic models and indicates the available soil water for plants. Both *L* and *θ* can be estimated by ground-based measurements, remote sensing derivations, and simulation modeling [7], [23]. Ground-based (direct and indirect) methods are relatively accurate at the site level, but cumbersome, costly, and even destructive to conduct [7]. Remote sensing has become a time and cost effective tool for the detection of spatial and temporal changes in *L* and *θ* over a large (>10 km^2^) area [23], [24], but at small scales (<10 km^2^) detecting spatial and temporal variability in *L* and *θ* is quite challenging due to problems associated with accuracy, time, and cost [23], [25]. In this study, we used Bayesian kriging [26], a novel data-model fusion approach to quantify and understand the spatio-temporal dynamics of *L* and *θ*. In practice, a model parameter is unknown and often replaced by estimated value as if the estimated value is true, thus ignoring the associated uncertainty in parameter estimation. Bayesian inference treats a parameter as a random variable and incorporates uncertainty in predictions (posterior probability) based on a prior probability and a likelihood function derived from the probability model for the observed data. Therefore, more realistic estimates of the model parameters and prediction variance are obtained.

The main objectives of this study were (1) to quantify the spatio-temporal interaction of *L* and *θ*, and (2) to assess the governing processes of this interaction at the Susquehanna Shale Hills Critical Zone Observatory (SSHCZO). We asked: (1) what is driving the spatio-temporal patterns of *L* in this forested watershed? *We hypothesized that the spatio-temporal patterns of L are driven by spatial patterns of different species generated due to topography, soil type and hydrology*; and (2) are the spatio-temporal patterns of *L* controlling the spatio-temporal patterns of *θ*? *We hypothesized that the spatio-temporal patterns of L will strongly influence the spatio-temporal patterns of θ across the watershed*.

## Materials and Methods

### Study site

**Figure 1:**
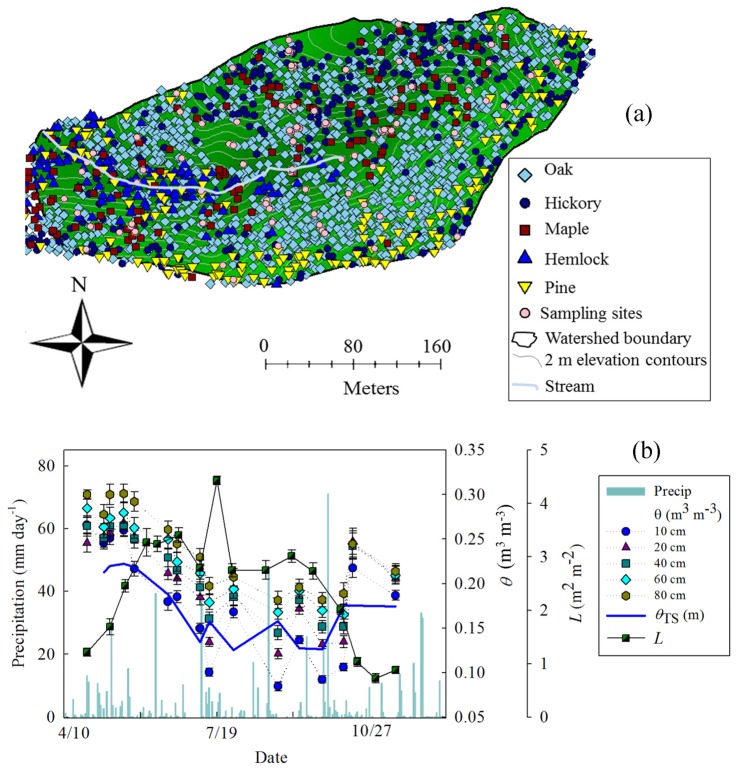
Map of the Susquehanna Shale Hills Critical Zone Observatory (a) showing spatial distribution of five dominant tree genera (Oak-*Quercus spp.*, Hickory-*Carya spp.*, Maple-*Acer spp*., Pine-*Pinus spp*., and Hemlock-*Tsuga sp.*) and spatial sampling grid across the watershed. (b) Measurements of leaf area index (*L*: m^2^ m^−2^), daily sum of precipitation, instantaneous volumetric water content (*θ*: m^3^ m^−3^) at varying soil depths (10–80 cm) and total profile soil moisture storage (*θ_TS_*: m) from April-November), 2010. Each point represents the mean of all sampled (∼90) points across watershed and vertical bars represent standard error of mean.

The Susquehanna Shale Hills Critical Zone Observatory (SSHCZO) spans over 7.9 ha in the Ridge and Valley region of central Pennsylvania. SSHCZO watershed is covered by approximately 110 years old humid temperate forest in which parent material is developing over a thick (>200 m) homogenous Rose Hill shale [27]. Five distinct soil series are present across the catchment including, Weikert (loamy-skeletal, mixed, active, mesic lithic dystrudepts), Berks (loamy-skeletal, mixed, active, mesic typic dystrudepts), Rushtown (loamy-skeletal, over fragmental, mixed, active, mesic typic dystrudepts), Ernest (fine-loamy, mixed, superactive, mesic aquic fragiudults), and Blairton (fine-loamy, mixed, active, mesic aquic hapludults) [28]. Most of the watershed is covered by deciduous trees, including maples (*Acer saccharum-*ACSA, *A. rubrum*-ACRU), hickories (*Carya cordiformis*-CACO, *C. glabra*-CAGL, *C. ovata*-CAOV, *C. tomentosa*-CATO), and oaks (*Quercus alba*-QUAL, *Q. prinus*-QUPR, *Q. rubra*-QURU, *Q. velatina*-QUVE). Conifer trees, including Eastern hemlock (*Tsuga canadensis*-TSCA)] and pines (*Pinus strobus*-PIST, *P. virginiana*-PIVI) also were fairly common in the catchment (Figure 1a and S1). The annual precipitation is ∼900 mm and the mean annual temperature is ∼11°C.

### Data Collection

A spatial sampling grid consisting of 90 sites (observed sites varied from 60–90 depending on weather) across the watershed was used to measure *L* and *θ*. The sampling grid was optimized to minimize measurement variability (nugget) and capture spatial variability by carefully choosing sites representing different landforms units (hilltop, hillslope, swale, and valley floor) and soils (Ernest, Blairton, Weikert, Berks, and Rushtown) in the catchment. Please see Lin 2006 [28] for detailed information about sampling design. The LI-2200 plant canopy analyzer (LI-COR, Inc., Lincoln, Nebraska, USA) was used to measure ground based forest *L*. The above canopy measurements were taken in an open space next to the forest area and below canopy measurements were taken at a predefined spatial sampling grid (Figure 1). Both above and below canopy measurements were taken as an average of four *L* measurements at each location with LI-2200 wand pointing in four (E, W, N, S) directions. A sunlit canopy was avoided by taking measurements in the early mornings, evenings or during overcast sky and a 45° restricted view of the sensor was used. Remotely sensed (MODIS) measurements of *L* every 8-day were obtained from ORNL-DAAC website (https://lpdaac.usgs.gov/get_data/) and rescaled for the site *L*.

A TRIME-FM Time Domain Reflectometry (TDR) device was used to collect volumetric soil water content (*θ*) at 10, 20, 40, 60, and 80 cm soil depths at sites co-located with *L* measurements by inserting the soil moisture probe into a PVC access tube buried at each site [28]. Total profile soil moisture storage (*θ_TS_*) for a particular site is calculated by the following equation [29]: 
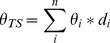
(1)


where *n* is the number of depth points available at a site, *θ_i_* is the volumetric soil moisture content at the *i* ^th^ depth and *d_i_* is the representative length of the *i* ^th^ depth interval. The depth interval length (*d*) was 0.15 m for 10 and 20 cm depths, and 0.20 m for 40, 60, 80 and 100 cm depths. Surface (10 cm) *θ* showed the most spatial and temporal variability [30], so the subsequent analyses were performed on the surface soil layer only.

Elevation and slope data were derived from a high-resolution 0.5×0.5 m DEM raster dataset for the SSHCZO, which was gathered by a LiDAR flight in February 2011 and was preprocessed at the University of California-Merced. TerraScan (Terrasolid: http://terrasolid.fi) software was used to classify the raw LiDAR point data into “bare-earth” and “above-ground” points. Ordinary kriging was used to interpolate the ground points and generate the digital elevation model (DEM) at 1 m resolution [31]. The relief between the highest and the lowest point across the watershed was 51.4 m. Slope value (radian) was calculated from the Shale Hills DEM using Maximum Triangle Slope method [32].

### Statistical Analyses

#### Lag Analysis

Remotely sensed canopy *L* data were used to gapfill ground based *L* data to match with *θ* when data were not collected on the same date. In addition linear interpolation was conducted to gapfill *L* and *θ* when concurrent data were missing. Regression analysis was performed between *L* and *θ* at different lags.

#### Spatial modeling

We used Bayesian kriging, a fully probabilistic Gaussian spatial model [26], [33], for spatial interpolation. A brief summary of modeling approach is given below and the detailed information can be found in [26]. Bayesian kriging assumes that observed data *Yi*: *i*  =  1,…,*n* are conditionally independent given a Gaussian underlying process *S* with:
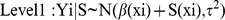


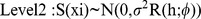


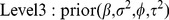
(2)


The first level describes a spatial linear trend (*β*  =  trend parameter) based on spatially referenced explanatory variables. The variance *τ^2^* (nugget) represents measurement variability and/or spatial variation below the sampling grain. The second level describes a stationary Gaussian spatial process [*S*(*xi*)] with mean  =  0, variance  =  *σ^2^* and correlation function R(*h*;*φ*), where *φ* is correlation parameter (range of spatial autocorrelation = 3*φ*) and *h* is lag distance (vector distance between two locations), and the third level specifies the prior for the model parameters. We chose an exponential correlation function:
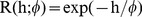
(3)


**Figure 2:**
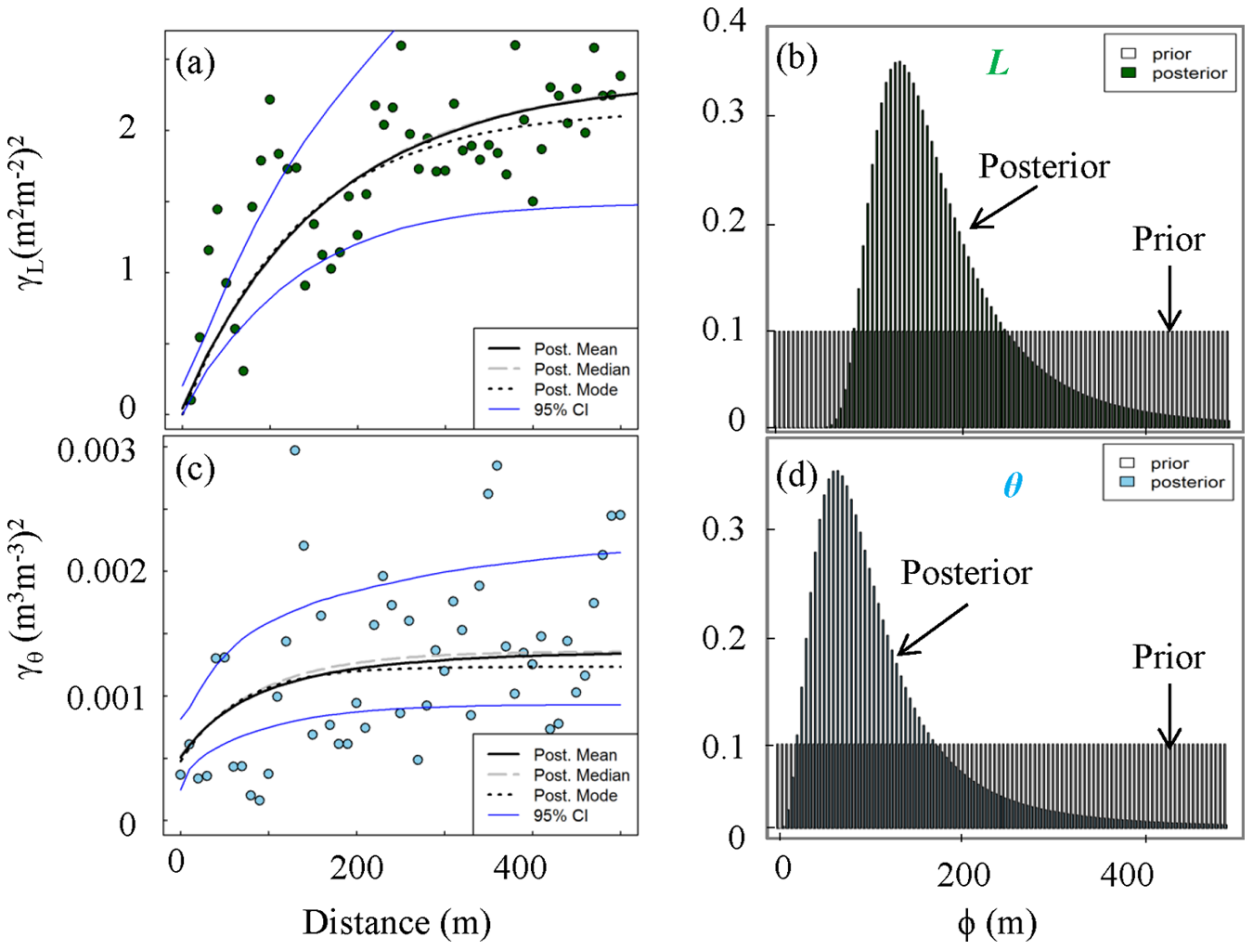
Representative semivariograms of (a) leaf area index (*L*: m^2^ m^−2^), and (b) volumetric water content (*θ* : m^3^ m^-3^) of surface (10 cm) soil. *γ* is semivariance and *φ* is range (3*φ*) parameter of spatial model. Discrete uniform prior and a discrete marginal posterior distribution of *φ* are displayed for (c) *L*, and (d) *θ*.

The mean and variance of *L* and *θ* were estimated at individual locations from the predictive distribution using the *krige.bayes* function of geoR library [34] in R version 2.15.0 [35]. This algorithm uses discrete distribution and parameter prior to compute the discrete posterior distribution and samples a parameter value from it. We assumed a constant trend mean model and used a multidimensional (100×100×100) parameter [*φ, σ^2^*, and *τ^2^.rel* (relative nugget = *τ^2^/σ^2^*)] grid by choosing a sensible interval of values for each parameter considering the study site. Please see R script (Script S1) for exact intervals for individual parameters. Flat prior (see Figure 2 for an example of prior and posterior distributions) were chosen for *φ*, and *τ^2^.rel*, and a reciprocal prior for *σ* ^2^. The sampled parameter value is then attached to [*β* | Y, *φ, σ^2^, τ^2^.rel*] and a realization is obtained from the predictive distribution at the desired location. This process was repeated several times so that the sample is large enough to permit stable estimation of the underlying distribution. The mean and the variance of the predictive distribution were computed at individual locations using 100,000 posterior draws. Leave-One-Out cross-validation strategy was used for model validation.

#### Density curves

To explore the distribution of different species across a gradient of elevation, slope and *θ*, smooth density curves, using *density* function in R, were calculated for LiDAR derived elevation and slope data, and spatially interpolated *θ* data at individual tree locations. Additionally, to explore the gain in *L* and loss of *θ* at individual tree location from budbreak to leaf maturity, density curves were calculated for spatially interpolated *L* and *θ* at individual tree locations. Tree location data were used as a prediction grid in Bayesian kriging for spatial prediction of *L* and *θ*.

## Results

**Figure 3:**
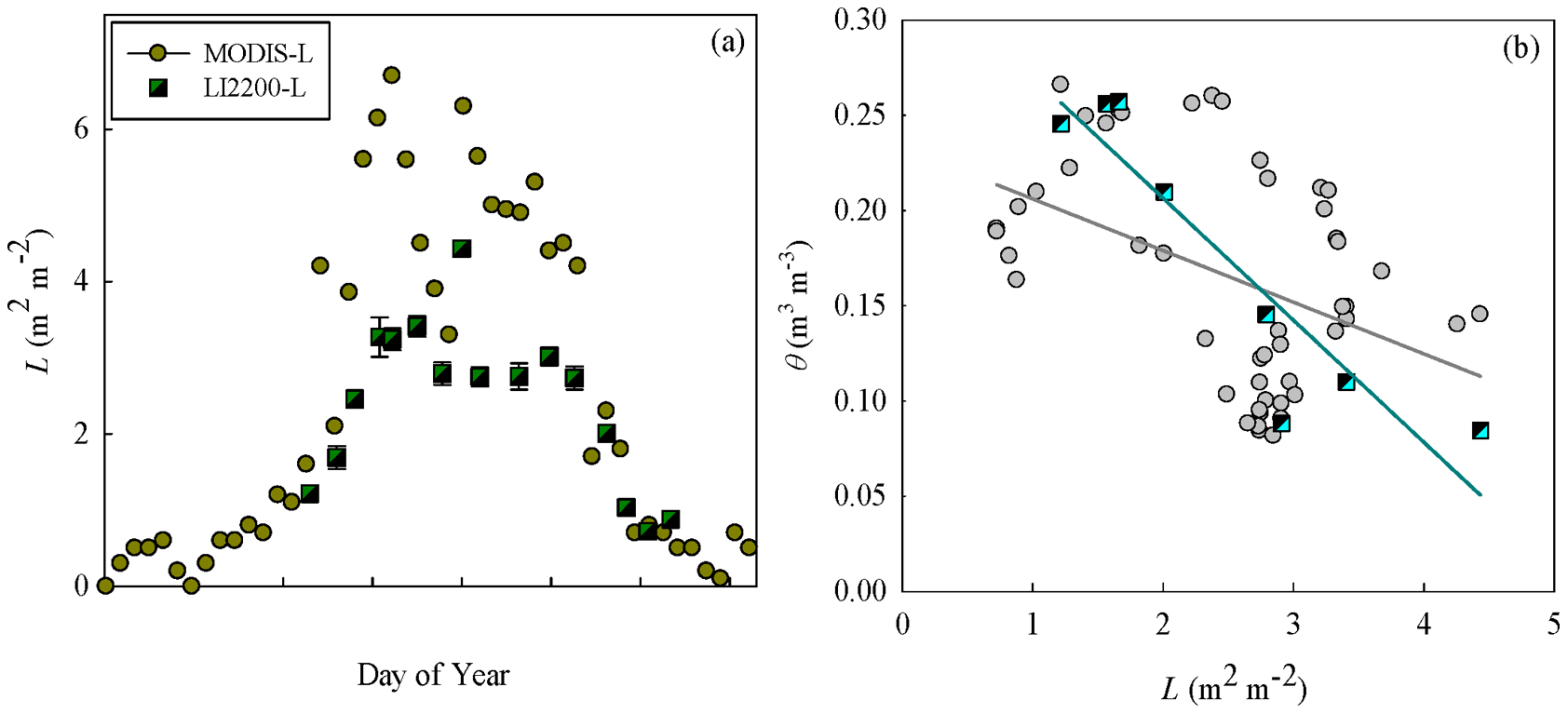
Relationship between (a) leaf area index (*L*: m^2^ m^−2^) observed from space (MODIS-*L*, 1 pixel for entire watershed) and ground (LI2200-*L*, average of ∼90 points across the watershed); and (b) *L* and volumetric water content (*θ*: m^3^ m^−3^) of surface soil. Filled circles represent the data at the same date and filled square represent 11 days lagged data.

The ground based observations (LI2200) and the remotely sensed (MODIS) *L* showed similar trends of budbreak, maturity and senescence (Figure 3a), but the *MODIS-L* was greater than the *LI2200-L. MODIS-L* was rescaled to fit the highest observed value of *LI2200-L* and zeros were replaced with linearly interpolated data. The surface (10 cm) *θ* explained the most variability in *L*, so all further analyses were performed on surface *θ*.

### What is driving the spatio-temporal patterns of *L* in this forested watershed?

**Figure 4:**
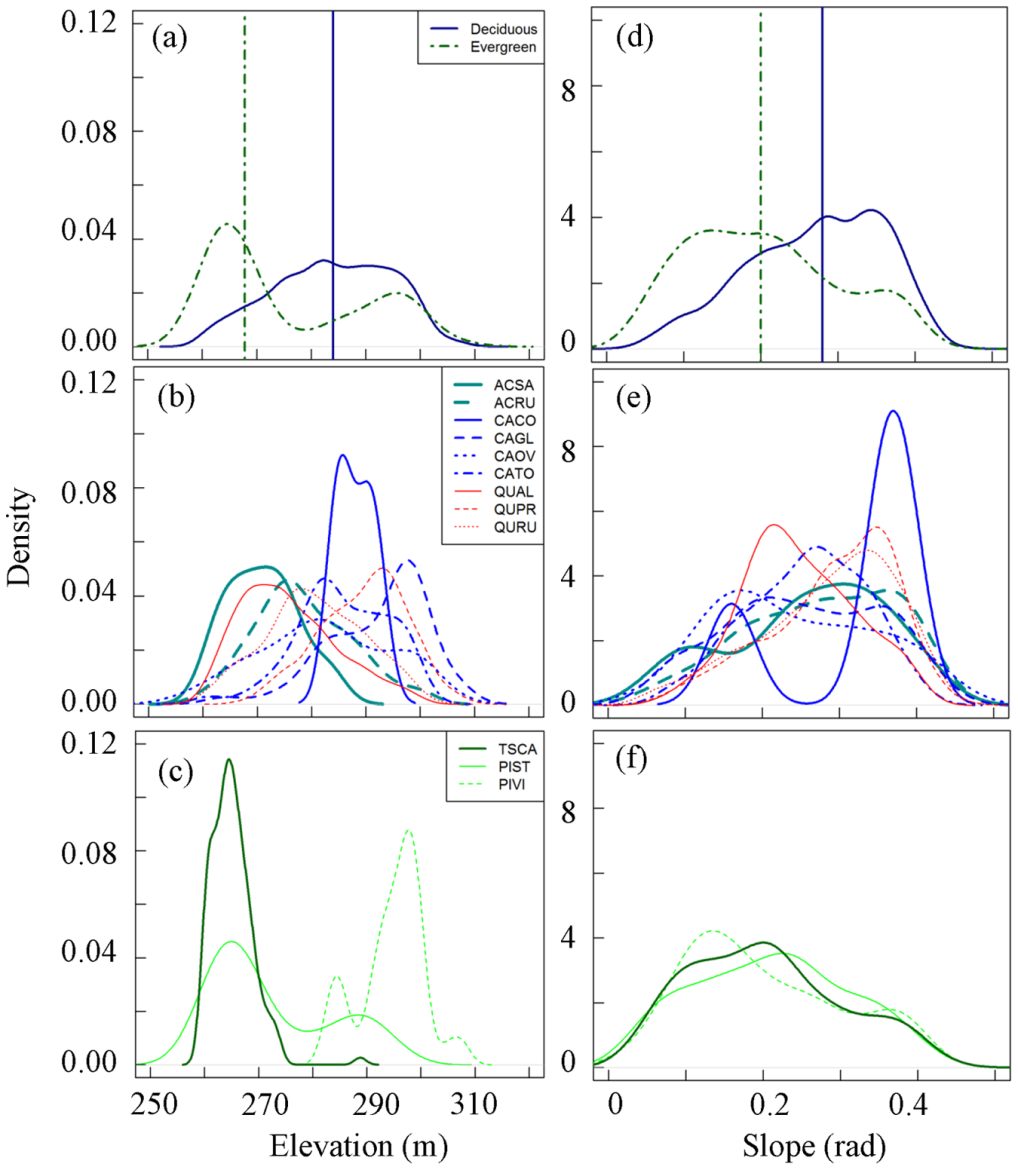
Distribution of deciduous (*Acer saccharum*-ACSA, *A. rubrum*-ACRU, *Carya cordiformis*-CACO, *C. glabra*-CAGL, *C. ovata*-CAOV, *C. tomentosa*-CATO, *Quercus alba*-QUAL, *Q. prinus*-QUPR, *Q. rubra*-QURU) and evergreen (*Tsuga canadensis*-TSCA, *Pinus strobus*-PIST, *P. virginiana*-PIVI) species across a gradient of (a–c) elevation, and (d–f) slope. Vertical lines represent the center (mode) of the distribution. Density curves were calculated from the values sampled at each tree location (total trees = 1832).

**Figure 5:**
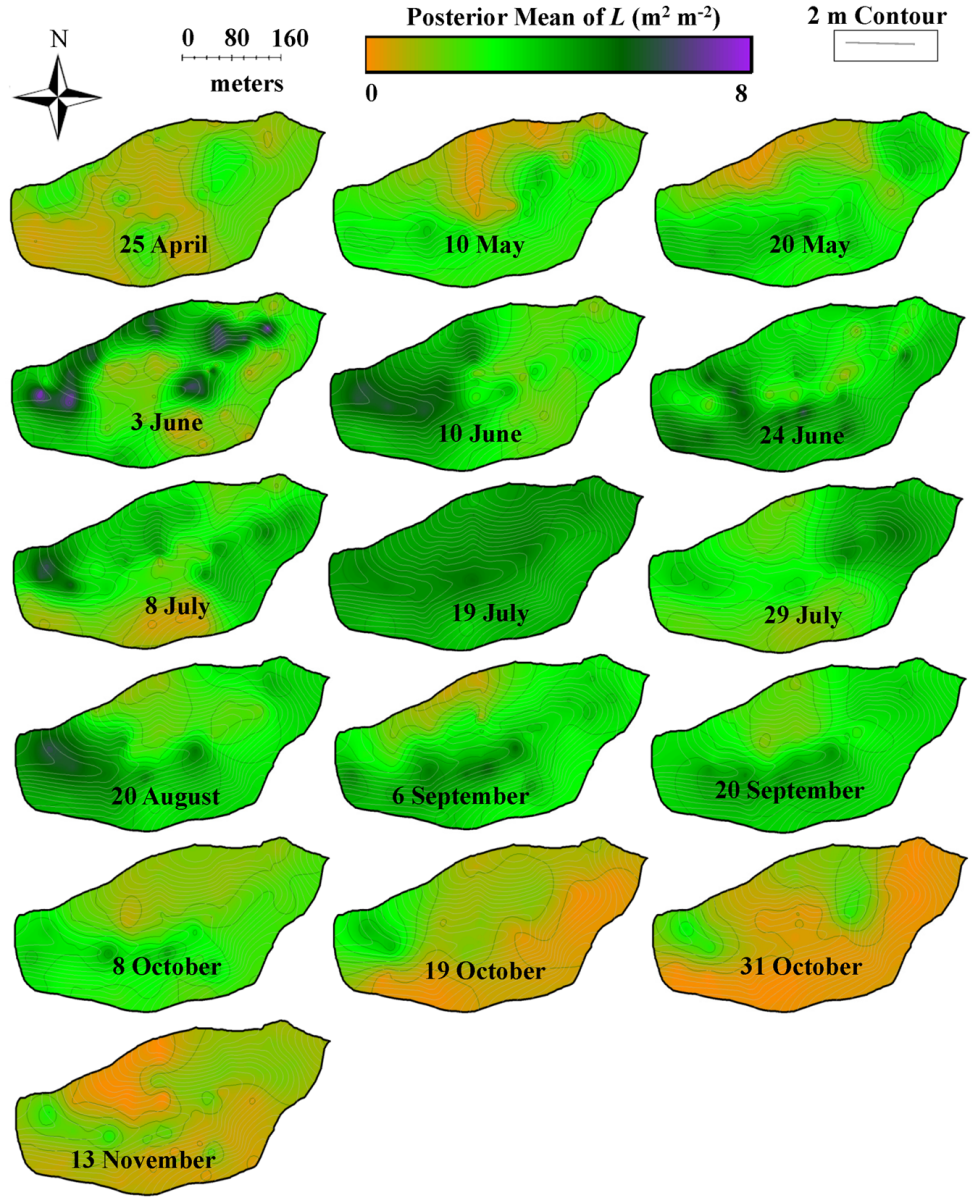
Temporal dynamics of leaf area index (*L*: m^2^ m^−2^) across Susquehanna Shale Hills CZO during 2010. Kriging was performed using hierarchical Bayesian model and maps of posterior mean are displayed.

Figure 1a shows the spatial distribution of dominating tree species across the watershed. Deciduous trees (oaks, hickories, and maples) are generally present at higher elevation (Figure 4) and in general avoid low slope locations (Figure 4), while evergreen trees (hemlock and pines) are concentrated on south ridge and south-west valley floor along the stream (Figure 1a), which have lowest slope across the watershed. Evergreen trees in general prefer lower elevation and lower slope locations (Figure 4a,d). At the species level, both maple species (ACSA and ACRU) occupied lower elevations while the four hickory species were found at moderate (CAOV and CACO) or higher (CAGL and CATO) elevations (Figure 4b). Oak species were present at low (QUAL), moderate (QURU), and high (QUPR) elevation (Figure 4b) making it the most abundant and widely distributed genus. Eastern hemlock (TSCA) and one of the pine species (PIST) were primarily present at low elevation (Figure 4c) and the wet valley floor alongside of the stream (Figure 1a), while the other pine species (PIVI) was distributed along the dry south ridge (Figure 1a). Soil water content did not significantly influence the spatial distribution of different species (Figure S2). Soil moisture content explained the occurrence of some species such as eastern hemlock (TSCA) and red maple (ACRU), which were restricted to the wettest region of the watershed along the stream, while mockernut hickory (CATO) and chestnut oak (QUPR) avoided the wetter regions of the watershed (Figure 1a, S2). Other species seemed to grow without any specific preference to a particular type of hydrologic regime, such as white, red and black oak (QUAL, QURU, QUVE), shagbark hickory (CAOV) and sugar maple (ACSA) (Figure S2). Eastern hemlock (TSCA) was restricted to the Ernest soil, red maple was found on Ernest and Rushtown soils, hickories and chestnut oak preferred Weikert soil and the rest of the species did not show any particular affinity to one type of soil. Despite the differences in elevation, soil type, and soil moisture, all evergreen species were present on relatively flat terrains (Figure 4f). The resulting spatial distribution and mixture of different species created unique spatio-temporal patterns of *L*, including timing of budbreak, maturity, and senescence. For instance, red maple (ACRU) showed earlier budburst, greater *L*, but similar senescence as red oak (Figure S3). The variability in leaf expansion (increase in *L*) of different species (Figure S2) also added complexity in spatial patterns of *L* resulting in a unique temporal pattern of *L* across the watershed (Figure 5).

### Are the spatio-temporal patterns of *L* controlling the spatio-temporal patterns of *θ*?

**Figure 6:**
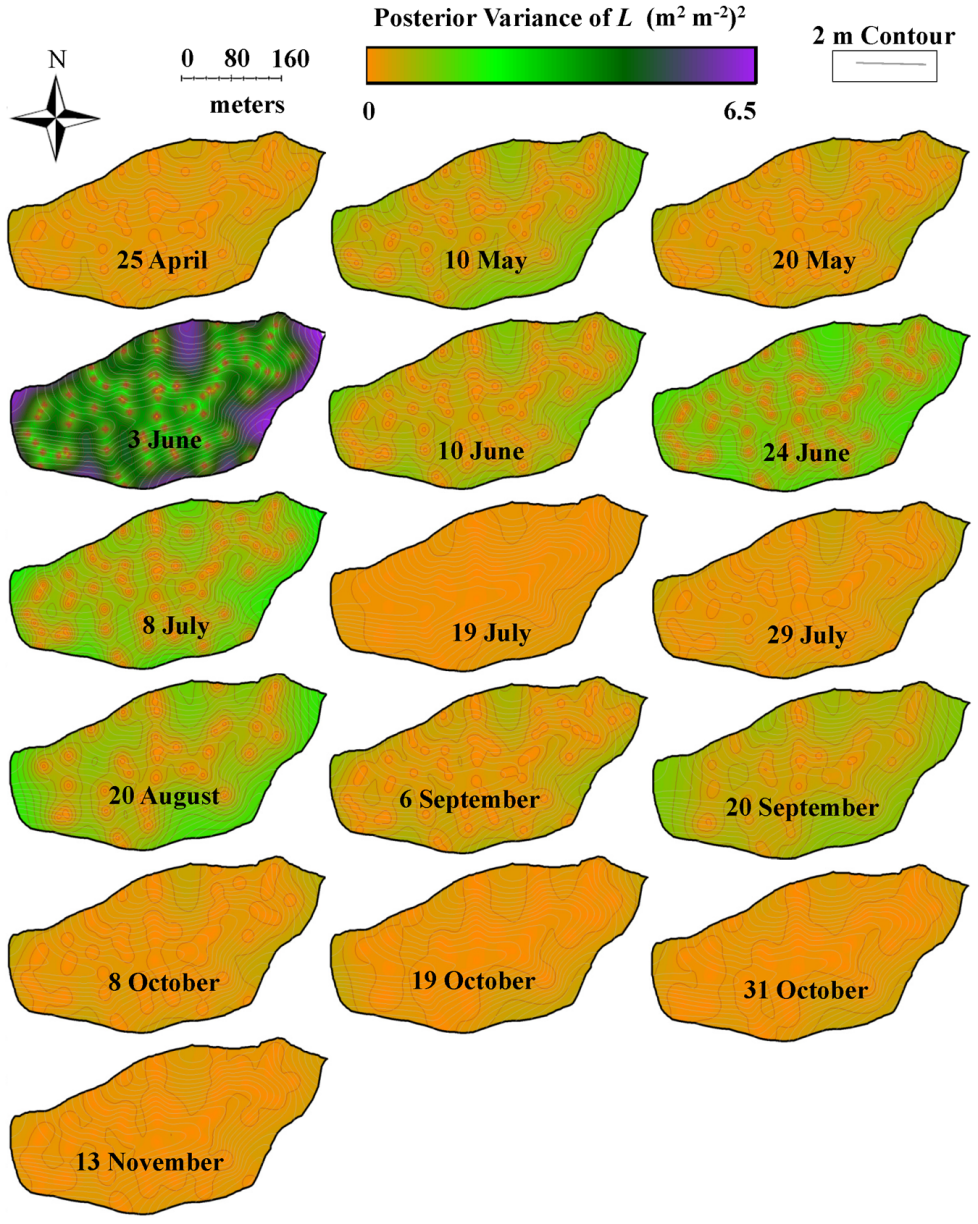
Temporal dynamics of posterior variance in leaf area index (*L*: m^2^ m^−2^) across Susquehanna Shale Hills CZO during 2010. Kriging was performed using hierarchical Bayesian model and maps of posterior variance are displayed.

**Figure 7:**
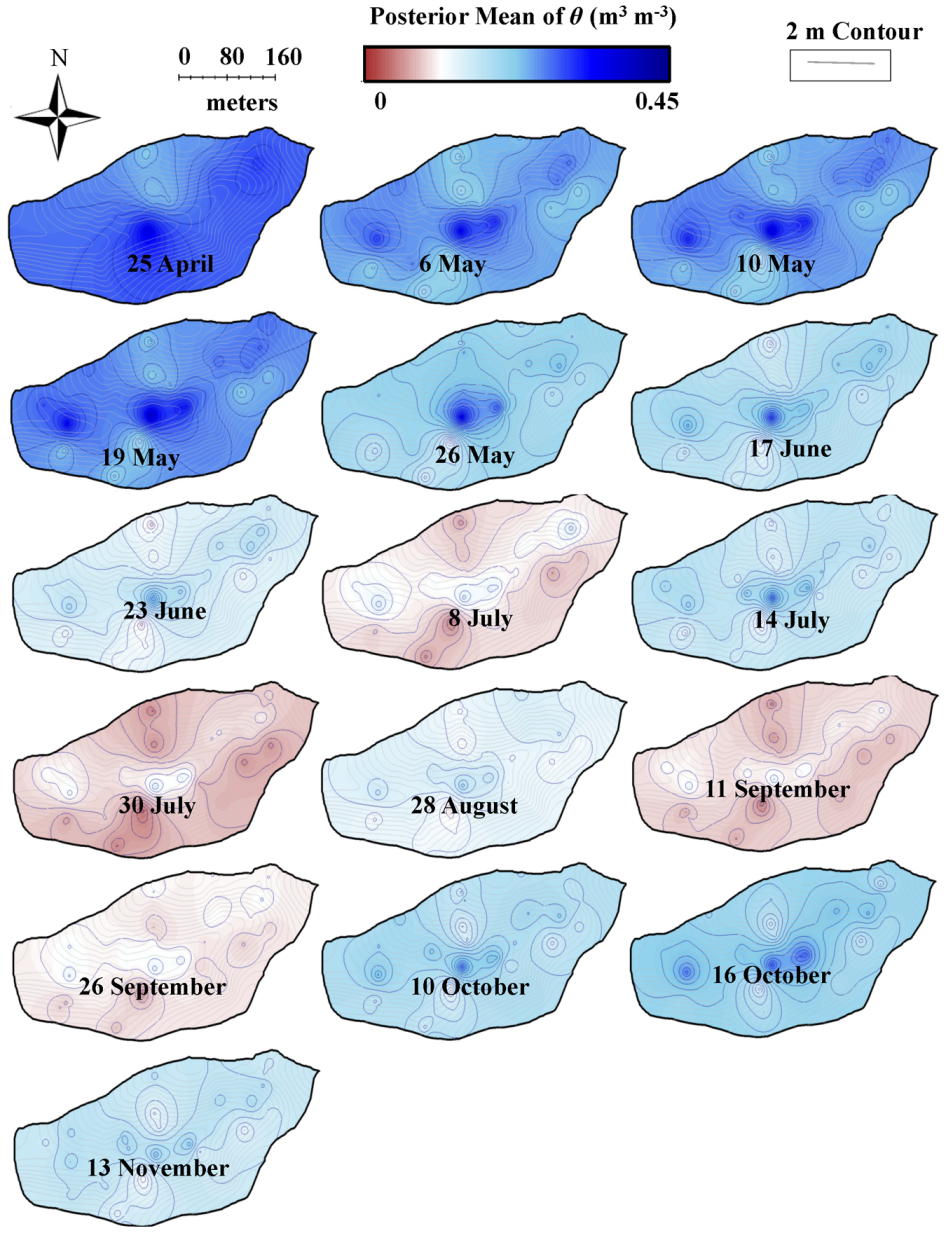
Temporal dynamics of surface (10 cm) volumetric soil water content (*θ*: m^3^ m^−3^) across Susquehanna Shale Hills CZO during 2010. Kriging was performed using hierarchical Bayesian model and maps of posterior mean are displayed.

**Figure 8:**
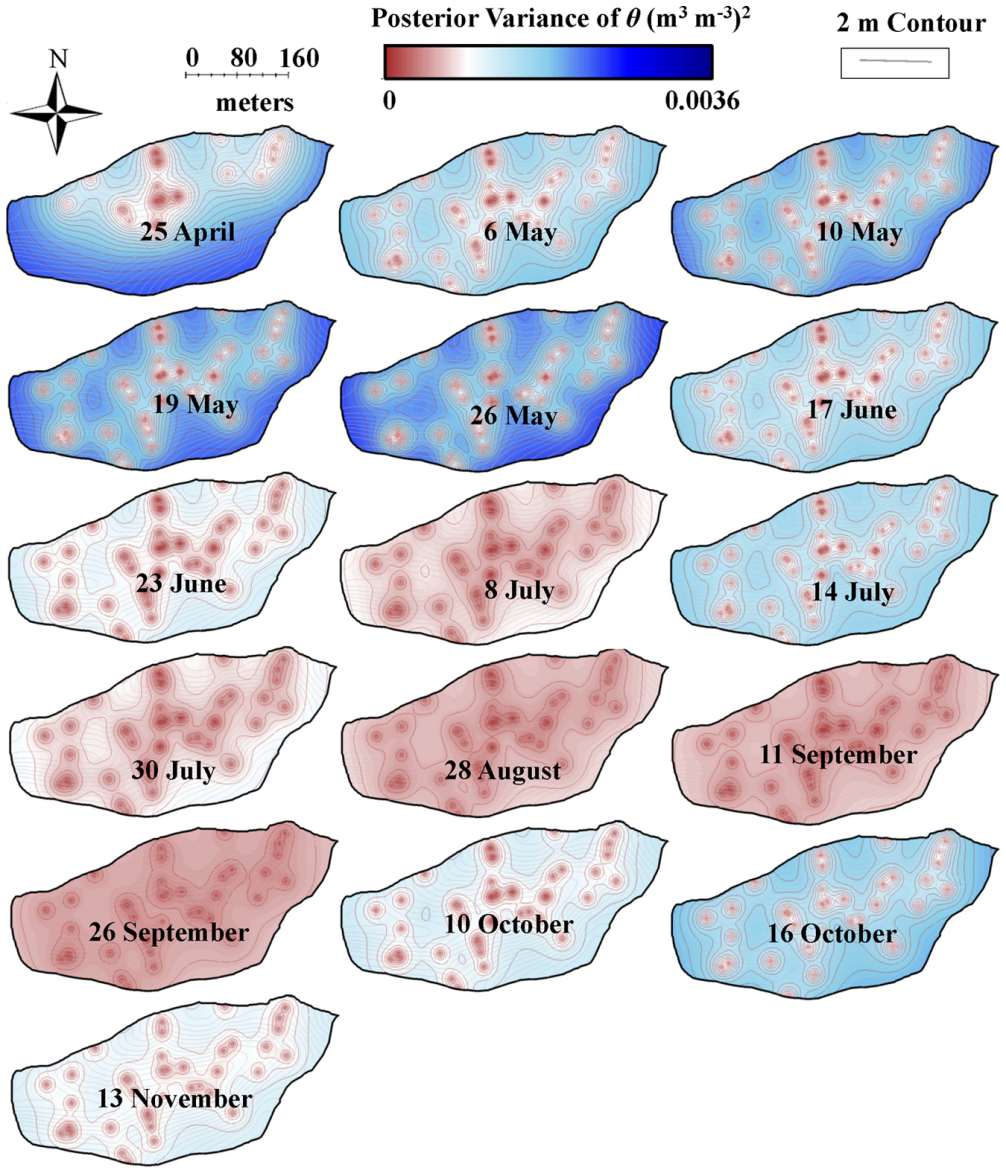
Temporal dynamics of surface (10 cm) volumetric soil water content (*θ*: m^3^ m^−3^) across Susquehanna Shale Hills CZO during 2010. Kriging was performed using hierarchical Bayesian model and maps of posterior variance are displayed.

*L* showed an exponential increase from April (leaf onset/budbreak) to July (leaf maturity) and reached the maximum in mid-July (leaf maturity) (19 July) (Figure 1b). Furthermore, an exponential decline in *L* was observed from July (leaf maturity) to November (senescence) (Figure 1b). An exponential decline in *θ* was observed at all measured soil depths (10–80 cm) and in total moisture storage (*θ_TS_*), which coincided with the exponential increase in *L*. Moreover, there was a subsequent rise in *θ* and *θ_TS_* with declining *L* (Figure 1b). *L* and *θ* were negatively correlated and their relationship showed hysteresis with ∼11-day lag between increase in *L* and decrease in *θ* (Figure 3b). The lower elevation species (both deciduous and evergreen) produced more leaf area and experienced less decline in surface soil moisture while higher elevation species produced less leaf area and experienced greater decline in soil moisture (Figure S2). The spatial mean of *L* across the watershed (mean of all modeled values of *L* in the watershed at 1×1 m grid) increased from leaf onset to maturity and then decreased from maturity to senescence (Figure 5), whereas spatial variability (standard error of spatial mean of *L*) and prediction variance were highest during budbreak and declined to a minimum during closed canopy and again increased from canopy closure to senescence (Figure 6). On the other hand, spatial variability and prediction variance of *θ* was correlated with the spatial mean of *θ* (Figure 7,8). Overall the kriged maps of *L* (Figure 5) and *θ* (Figure 7) confirm the temporal trend of instantaneous measurements.

**Figure 9:**
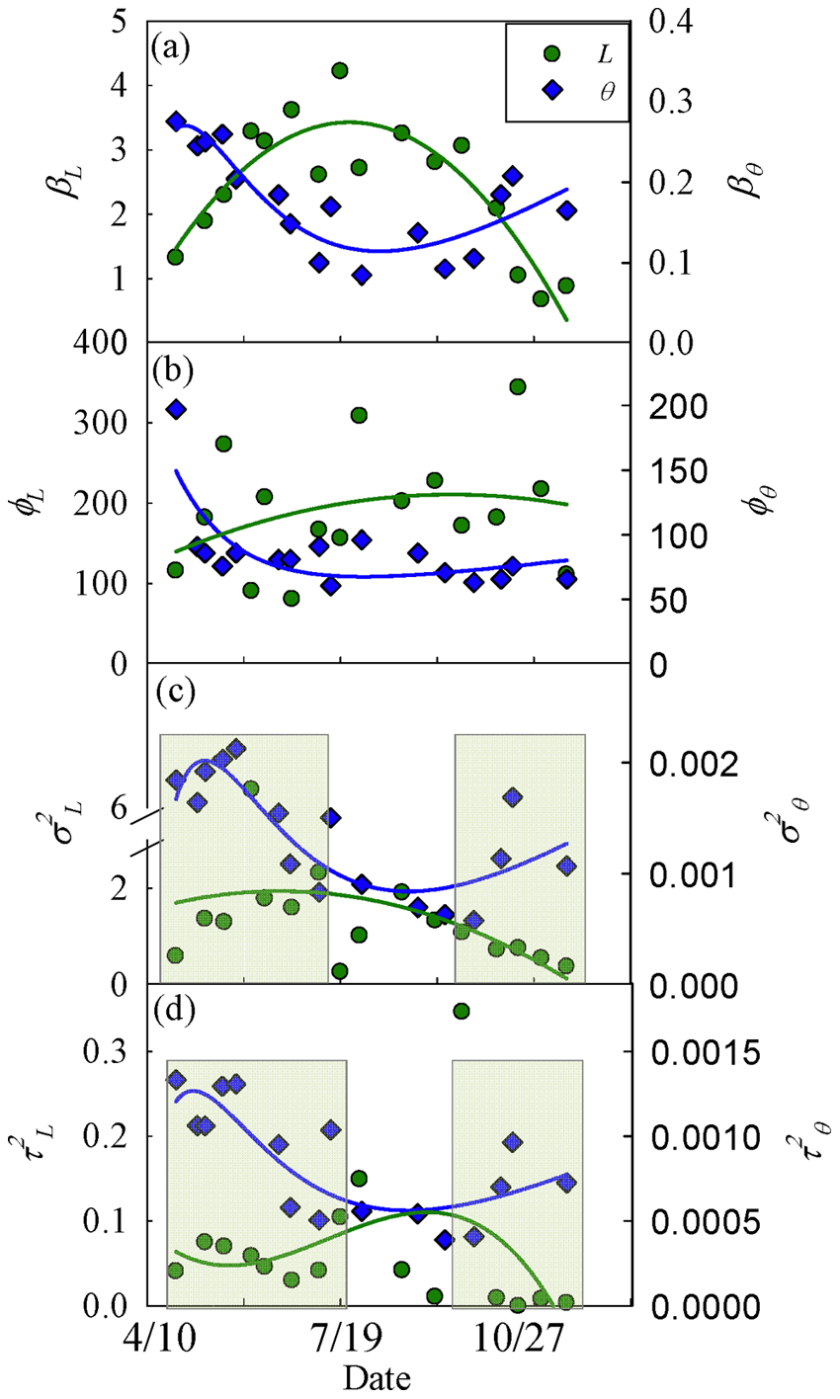
Temporal dynamics of spatial model parameters of leaf area index (*L*: m^2^ m^−2^) and volumetric soil (10 cm) water content (*θ*: m^3^ m^−3^) from April–November, 2010. *β* is trend parameter, *φ* is range parameter (range = 3*φ*), *σ* ^2^ is partial sill, and *τ* ^2^ is nugget. Each point represents the posterior mean of an estimated parameter for one date and solid line represents the fitted curve. Shaded regions mark budbreak and senescence periods when spatial structure [nugget (*τ* ^2^) and sill (*σ* ^2^)] of *L* and *θ* was uncoupled.

The measurement error (nugget, *τ* ^2^) and the spatial variance (sill, *σ* ^2^) of *L* and *θ* increased with increasing spatial mean of *L* and *θ* (Table 1,2). This relationship was also reflected in the inverse temporal trends of spatial structure (spatial model parameters-*β, φ, τ^2^, σ^2^*) of *L* and *θ* (Figure 9a–d) similar to the inverse relationship of their mean values (Figure 1b). The nugget and sill of *L* increased from leaf onset (April) to maturity (July) and then decreased from leaf maturity to senescence (November), while the nugget and the sill of *θ* decreased from leaf onset to maturity and then increased from leaf maturity to senescence (Figure 9a–d). Leave-one-out cross-validation showed good agreement between observed and predicted values for both *L* (R^2^ = 0.92 −0.99) and *θ* (R^2^ = 0.76–0.96) (Figure S4, S5).

**Table 1:** Summary of semivariogram parameters estimated from Bayesian model for leaf area index (*L*: m^2^ m^−2^).

Date	*β* (Trend)	*φ* (Range = 3*φ*)	*σ* ^2^ (Sill)	*τ* ^2^ (Nugget)	*τ* ^2^/(*τ* ^2^+*σ* ^2^) (NSR)	R^2^
4/25/2010	1.33 (0.94, 1.71)	116.16 (70.71, 232.32)	0.60 (0.41, 1.03)	0.04 (0.02, 0.09)	0.06 (0.02, 0.17)	0.93
5/10/2010	1.89 (1.01, 2.70)	181.82 (95.96, 388.89)	1.37 (0.85, 2.59)	0.07 (0.02, 0.19)	0.05 (0.01, 0.17)	0.97
5/20/2010	2.30 (1.36, 3.17)	272.73 (151.52, 454.55)	1.30 (0.81, 2.21)	0.07 (0.03, 0.13)	0.05 (0.02, 0.13)	0.99
6/3/2010	3.28 (2.15, 4.29)	90.91 (60.61, 166.67)	6.13 (4.36, 9.93)	0.06 (0.00, 0.45)	0.01 (0.00, 0.08)	0.95
6/10/2010	3.13 (2.13, 4.01)	207.07 (126.26, 388.89)	1.79 (1.17, 3.22)	0.05 (0.00, 0.13)	0.02 (0.00, 0.09)	0.98
6/24/2010	3.61 (3.12, 4.12)	80.81 (55.56, 141.41)	1.60 (1.15, 2.44)	0.03 (0.00, 0.14)	0.02 (0.00, 0.09)	0.98
7/8/2010	2.61 (1.59, 3.52)	166.67 (95.96, 353.54)	2.32 (1.52, 4.41)	0.04 (0.00, 0.16)	0.02 (0.00, 0.08)	0.98
7/19/2010	4.22 (3.75, 4.54)	156.57 (60.61, 383.84)	0.27 (0.16, 0.55)	0.10 (0.06, 0.17)	0.38 (0.13, 0.88)	0.92
7/29/2010	2.72 (1.85, 3.53)	308.08 (176.77, 469.70)	1.02 (0.63, 1.73)	0.15 (0.09, 0.24)	0.14 (0.06, 0.31)	0.96
8/20/2010	3.25 (2.26, 4.30)	202.02 (116.16, 404.04)	1.92 (1.17, 3.59)	0.04 (0.00, 0.17)	0.02 (0.00, 0.11)	0.97
9/6/2010	2.81 (1.90, 3.57)	227.27 (131.31, 424.24)	1.33 (0.84, 2.47)	0.01 (0.00, 0.06)	0.01 (0.00, 0.05)	0.99
9/20/2010	3.06 (2.32, 3.80)	171.72 (85.86, 378.79)	1.08 (0.63, 2.07)	0.35 (0.21, 0.56)	0.31 (0.13, 0.72)	0.93
10/8/2010	2.09 (1.52, 2.64)	181.82 (111.11, 358.59)	0.73 (0.48, 1.34)	0.01 (0.00, 0.04)	0.01 (0.00, 0.06)	0.99
10/19/2010	1.05 (0.27, 1.80)	343.43 (202.02, 484.85)	0.76 (0.47, 1.22)	0.00 (0.00, 0.02)	0.00 (0.00, 0.02)	0.99
10/31/2010	0.68 (0.09, 1.21)	217.17 (121.21, 419.19)	0.55 (0.34, 1.03)	0.01 (0.00, 0.03)	0.01 (0.00, 0.05)	0.99
11/13/2010	0.88 (0.57, 1.17)	111.11 (70.71, 207.07)	0.38 (0.27, 0.65)	0.00 (0.00, 0.02)	0.01 (0.00, 0.06)	0.99

**Notes:** NSR is noise to signal ratio and R^2^ is linear model fit of leave-one-out cross validation between modeled and observed *L*. Parameter estimates represent mean of posterior distribution and values inside the parenthesis are quantile based 5% and 95% credible intervals.

**Table 2:** Summary of semivariogram parameters estimated from Bayesian model for surface (10 cm) soil water content (*θ*: m^3^ m^−3^).

Date	*β* (Trend)	*φ* (Range = 3*φ*)	*σ* ^2^ (Sill)	*τ* ^2^ (Nugget)	*τ* ^2^/(*τ* ^2^+*σ* ^2^) (NSR)	R^2^
4/25/2010	0.28 (0.24, 0.32)	196.97 (45.45, 449.49)	0.0018 (0.0012, 0.0035)	0.0013 (0.0009, 0.0020)	0.76 (0.38, 0.98)	0.76
5/6/2010	0.24 (0.22, 0.27)	90.91 (35.35, 252.53)	0.0016 (0.0011, 0.0029)	0.0011 (0.0007, 0.0015)	0.67 (0.31, 0.97)	0.81
5/10/2010	0.25 (0.23, 0.27)	85.86 (40.40, 207.32)	0.0019 (0.0013, 0.0035)	0.0011 (0.0007, 0.0016)	0.56 (0.24, 0.94)	0.83
5/19/2010	0.26 (0.23, 0.28)	75.76 (30.30, 207.07)	0.0020 (0.0014, 0.0036)	0.0013 (0.0009, 0.0019)	0.66 (0.30, 0.96)	0.83
5/26/2010	0.20 (0.18, 0.23)	85.86 (30.30, 262.63)	0.0021 (0.0014, 0.0037)	0.0013 (0.0008, 0.0020)	0.64 (0.27, 0.96)	0.89
6/17/2010	0.18 (0.16, 0.20)	80.81 (30.30, 212.12)	0.0015 (0.0010, 0.0027)	0.0010 (0.0006, 0.0014)	0.63 (0.28, 0.96)	0.84
6/23/2010	0.15 (0.13, 0.16)	80.81 (30.30, 207.07)	0.0011 (0.0007, 0.0019)	0.0006 (0.0003, 0.0009)	0.54 (0.22, 0.93)	0.85
7/8/2010	0.10 (0.08, 0.11)	90.91 (30.30, 292.93)	0.0008 (0.0005, 0.0015)	0.0005 (0.0003, 0.0008)	0.63 (0.24, 0.96)	0.86
7/14/2010	0.17 (0.15, 0.19)	60.61 (20.20, 176.77)	0.0015 (0.0010, 0.0025)	0.0010 (0.0007, 0.0015)	0.72 (0.34, 0.98)	0.86
7/30/2010	0.08 (0.07, 0.10)	95.96 (35.35, 303.03)	0.0009 (0.0006, 0.0017)	0.0006 (0.0003, 0.0008)	0.64 (0.25, 0.97)	0.85
8/28/2010	0.14 (0.12, 0.15)	85.86 (20.20, 358.59)	0.0007 (0.0005, 0.0012)	0.0005 (0.0004, 0.0008)	0.81 (0.44, 0.99)	0.86
9/11/2010	0.09 (0.08, 0.10)	70.71 (25.25, 237.37)	0.0006 (0.0004, 0.0011)	0.0004 (0.0002, 0.0006)	0.64 (0.23, 0.97)	0.84
9/26/2010	0.11 (0.09, 0.12)	63.13 (10.10, 384.09)	0.0006 (0.0004, 0.0010)	0.0004 (0.0002, 0.0007)	0.76 (0.26, 0.99)	0.94
10/10/2010	0.18 (0.17, 0.20)	65.66 (25.25, 161.62)	0.0011 (0.0008, 0.0019)	0.0007 (0.0004, 0.0011)	0.64 (0.27, 0.96)	0.86
10/16/2010	0.21 (0.18, 0.23)	75.76 (20.20, 287.88)	0.0017 (0.0011, 0.0031)	0.0010 (0.0002, 0.0017)	0.62 (0.09, 0.97)	0.96
11/13/2010	0.16 (0.15, 0.18)	65.66 (15.15, 313.13)	0.0011 (0.0007, 0.0019)	0.0007 (0.0003, 0.0012)	0.73 (0.24, 0.98)	0.94

**Notes:** NSR is noise to signal ratio and R^2^ is linear model fit of leave-one-out cross validation between modeled and observed *θ*. Parameter estimates represent mean of posterior distribution and values inside the parenthesis are quantile based 5% and 95% credible intervals.

## Discussion

### What is driving the spatio-temporal patterns of L in this forested watershed?

Variation in phenology and factors influencing it have attracted the attention of ecologists for a long time and current literature shows that multiple factors, such as hydrology [36], [37], variation in leaf longevity [38], [39], tree height [40], CO_2_ concentration and nutrients [41], temperature [42]–[44] and light availability [45] greatly influence leaf phenology. However, influence of biodiversity on coupled dynamics of phenology and hydrology across a landscape is largely untested. Our results support the first hypothesis partially and show that spatial distribution of tree species drives the spatio-temporal patterns of *L* in the watershed and depends on topography and soil type. However, soil hydrology was not a good predictor of species distribution across the watershed, possibly due to humid conditions. Topography, mainly elevation and slope, greatly influenced the spatial distribution of different tree species across the watershed (Figure 2), which created a unique spatial pattern of leaf area index. Surprisingly, soil moisture did not explain the distribution of the tree species across the watershed (Figure S2). Soil type and slope explained the spatial distribution better than *θ*, except for eastern hemlock which was only present on wet soil along the stream. But the presence of TSCA can be better explained by soil type than hydrology. Furthermore, spatial pattern of *L* exhibited strong temporal dynamics due to different timings of budbreak, maturity, and senescence of leaves and variability in leaf expansion of different species, thus creating a spatially explicit forest phenology (Figure 5).

### Are the spatio-temporal patterns of L controlling the spatio-temporal patterns of θ?

Quantifying spatio-temporal patterns of *L* and *θ* is becoming increasingly important, as spatially distributed approaches become more common in current and future landscape modeling [16], [46]. Our results support our second hypothesis and show the coupled dynamics of *L* and *θ* in spatial and temporal domains. The spatial structure of leaf phenology and hydrology showed tight coupling at the peak of the growing season (closed canopy) (Figure 9), presumably primarily through evapotranspiration, as leaves control loss of water from plants through transpiration [3]–[5]. In addition, *L* likely influences loss of water from shallow soil layers (evaporation) through changing surface albedo and roughness [1]. At budbreak and senescence the spatial structure of *L* and *θ* were not coupled (Figure 9, shaded region) and water was distributed more uniformly (Figure 7) throughout the watershed (with variation mostly associated with the topographic complexity). *L* and *θ* showed a clear seasonal pattern and supported the previous findings of Takagi and Lin [30], which suggests greater control of evapotranspiration on soil moisture under dry conditions and topography under wet conditions. However, we found a lag of about 11 days between the increase in *L* and decline in *θ*, which could be due to the delay in full photosynthetic activity after leaf onset [47], [48], water storage inside tree stem [48]–[50], and soil moisture buffer zone around trees due to lateral water flow in soil [49], [51] and plant-aided hydraulic redistribution [8]–[10]. The temporal variation of vegetation and hydrology coupling was also visible in spatial structure of *L* and *θ* across the watershed (Figure 9). At budbreak and senescence the forest canopy was patchy and highly variable for *L* across the watershed due to differences in phenology of different species and *θ* showed high variability due to complex terrain [30] and variable demand of water from emerging and senescing leaves (Figure 5,7). At leaf maturity, forest canopy was closed (maximum *L*) and the spatial variability of *L* was minimum (Figure 5,6), while soil moisture (after 11 days) was very low and relatively uniformly distributed across the watershed and thus showed least variability (Figure 7,8).

## Conclusions

Results from this study suggest that spatial distribution of tree species and different timing of budbreak, maturity, and senescence for different species across the forested landscape created unique spatio-temporal patterns of *L*, which created the patterns of water demands reflected in variable soil water content in space and time. The landscape canopy and soil water became increasingly homogenized and coupled from leaf onset to maturity (i.e., increasing and homogenous *L*, and decreasing and homogenous *θ*), but became more heterogeneous and uncoupled from leaf maturity to senescence (i.e., patchy and decreasing *L*, and patchy and increasing *θ*). Our results provide insight into tight coupling between biodiversity and soil hydrology across space and time. Incorporating these spatial and temporal feedbacks into hydrologic models will improve current and future landscape modeling of humid temperate forests.

## Supporting Information

Figure S1 **Spatial distribution of deciduous (oaks [** ***Quercus alba-*** **QUAL,** ***Q. prinus-*** **QUPR,** ***Q. rubra-*** **QURU,** ***Q. velatina*** **-QUVE], hickories [** ***Carya cordiformis*** **-CACO,** ***C. glabra-*** **CAGL,** ***C. ovata*** **-CAOV,** ***C. tomentosa*** **CATO], maples [** ***Acer saccharum-*** **ACSA,** ***A. rubrum*** **-ACRU]) and conifer (pines [** ***Pinus strobus*** **-PIST,** ***P. virginiana*** **-PIVI] and eastern hemlock [** ***Tsuga canadensis-*** **TSCA]) trees across the Susquehanna Shale Hills Critical Zone Observatory.**
(TIF)

Figure S2 **Distribution of deciduous (*Acer saccharum*-ACSA, *A. rubrum*-ACRU, *Carya cordiformis*-CACO, *C. glabra*-CAGL, *C. ovata*-CAOV, *C. tomentosa*-CATO, *Quercus alba*-QUAL, *Q. prinus*-QUPR, *Q. rubra*-QURU) and evergreen (Tsuga canadensis-TSCA, Pinus strobus-PIST, P. virginiana-PIVI) species across a gradient of (a–c) time averaged volumetric soil (10 cm) water content (θ_average_: m^3^ m^−3^), (d–f) change in θ from budburst to closed canopy, 11 day were added to closed canopy to account for the lag between L and θ; and (g–i) change in leaf area index (L: m^2^ m^−2^) from budburst to closed canopy.** Vertical lines represent the center (mode) of the distribution. Density curves were calculated from the values sampled at each tree location (total trees = 1832).
(TIF)

Figure S3 **Examples of different timing of budburst, maturity and senescence in (a) deciduous (maple [** ***Acer rubrum-*** **ACRU] and oak [** ***Quercus prinus-*** **QUPR], and (b) evergreen (eastern hemlock [** ***Tsuga canadensis-*** **TSCA] and pine [** ***Pinus virginiana-*** **PIVI] trees.** Each point is an average of posterior mean of leaf area index (*L*: m^2^ m^−2^) for all trees within a species across the landscape and error bar represents standard error of mean.
(TIF)

Figure S4 **Figure showing leave-one-out cross-validation to assess the model goodness of fit.** Dotted line represents 1:1 line and solid line is the slope of linear regression between observed and modeled value of Leaf area index (*L*: m^2^ m^−2^).
(TIF)

Figure S5 **Figure showing leave-one-out cross-validation to assess the model goodness of fit.** Dotted line represents 1:1 line and solid line is the slope of linear regression between observed and modeled value of surface (10 cm) soil water content (*θ*: m^3^ m^−3^).
(TIF)

Script S1 **Example data and R script used in this paper.**
(ZIP)

## References

[1] Pielke RA, Avissar RI, Raupach M, Dolman AJ, Zeng X, et al (2003) Interactions between the atmosphere and terrestrial ecosystems: influence on weather and climate. Global Change Biology 4: 461–475.

[2] Crockford RH, Richardson DP (2000) Partitioning of rainfall into throughfall, stemflow and interception: effect of forest type, ground cover and climate. Hydrological Processes 14: 2903–2920.

[3] Stogsdill Jr W, Wittwer R, Hennessey T, Dougherty P (1989) Relationship between throughfall and stand density in a Pinus taeda plantation. Forest Ecology and Management 29: 105–113.

[4] Kikuzawa K (1995) Leaf phenology as an optimal strategy for carbon gain in plants. Canadian Journal of Botany 73: 158–163.

[5] Oren R, Ewers BE, Todd P, Phillips N, Katul G (1998) Water balance delineates the soil layer in which moisture affects canopy conductance. Ecological Applications 8: 990–1002.

[6] Tabacchi E, Lambs L, Guilloy H, Planty-Tabacchi AM, Muller E, et al (2000) Impacts of riparian vegetation on hydrological processes. Hydrological Processes 14: 2959–2976.

[7] Guo X, Si BC (2008) Characterizing LAI spatial and temporal variability using a wavelet approach. The International Archives of the Photogrammetry, Remote Sensing and Spatial Information Sciences Vol XXXVII Part B.

[8] Richards JH, Caldwell MM (1987) Hydraulic lift: Substantial nocturnal water transport between soil layers by Artemisia tridentata roots. Oecologia 73: 486–489. 28311963 10.1007/BF00379405

[9] Brooks JR, Meinzer FC, Warren JM, Domec J-C, Coulombe R (2006) Hydraulic redistribution in a Douglas-fir forest: lessons from system manipulations. Plant Cell Environ 29: 138–150. 17086760 10.1111/j.1365-3040.2005.01409.x

[10] Naithani KJ, Ewers BE, Pendall E (2012) Sap flux-scaled transpiration and stomatal conductance response to soil and atmospheric drought in a semi-arid sagebrush ecosystem. Journal of Hydrology 464–465: 176–185.

[11] Asbjornsen H, Vogt KA, Ashton MS (2004) Synergistic responses of oak, pine and shrub seedlings to edge environments and drought in a fragmented tropical highland oak forest, Oaxaca, Mexico. Forest ecology and management 192: 313–334.

[12] Booth MS, Caldwell MM, Stark JM (2003) Overlapping resource use in three Great Basin species: implications for community invasibility and vegetation dynamics. Journal of Ecology 91: 36–48.

[13] Breshears DD, Barnes FJ (1999) Interrelationships between plant functional types and soil moisture heterogeneity for semiarid landscapes within the grassland/forest continuum: a unified conceptual model. Landscape Ecology 14: 465–478.

[14] Choler P, Sea W, Briggs P, Raupach M, Leuning R (2010) A simple ecohydrological model captures essentials of seasonal leaf dynamics in semi-arid tropical grasslands. Biogeosciences 7: 907–920.

[15] Asbjornsen H, Goldsmith GR, Alvarado-Barrientos MS, Rebel K, Van Osch FP, et al (2011) Ecohydrological advances and applications in plant–water relations research: a review. Journal of Plant Ecology 4: 3–22.

[16] Vivoni ER (2012) Spatial patterns, processes and predictions in ecohydrology: integrating technologies to meet the challenge. Ecohydrology 5: 235–241.

[17] Breshears DD, Myers OB, Barnes FJ (2009) Horizontal heterogeneity in the frequency of plant-available water with woodland intercanopy–canopy vegetation patch type rivals that occuring vertically by soil depth. Ecohydrology 2: 503–519.

[18] Duniway MC, Snyder KA, Herrick JE (2010) Spatial and temporal patterns of water availability in a grass–shrub ecotone and implications for grassland recovery in arid environments. Ecohydrology 3: 55–67.

[19] Potts DL, Scott RL, Bayram S, Carbonara J (2010) Woody plants modulate the temporal dynamics of soil moisture in a semi-arid mesquite savanna. Ecohydrology 3: 20–27.

[20] Sellers PJ, Dickinson RE, Randall DA, Betts AK, Hall FG, et al (1997) Modeling the Exchanges of Energy, Water, and Carbon Between Continents and the Atmosphere. Science 275: 502–509. 8999789 10.1126/science.275.5299.502

[21] Chen JM, Black TA (1992) Defining leaf area index for non-flat leaves. Plant, Cell & Environment 15: 421–429.

[22] Sprintsin M, Karnieli A, Berliner P, Rotenberg E, Yakir D, et al (2007) The effect of spatial resolution on the accuracy of leaf area index estimation for a forest planted in the desert transition zone. Remote Sensing of Environment 109: 416–428.

[23] Lin H, Bouma J, Wilding LP, Richardson JL, Kutílek M, et al. (2005) Advances in Hydropedology Academic Press, Vol. Volume 85 . pp. 1–89. Available: http://www.sciencedirect.com/science/article/B7CSX-4FF2V9H-4/2/ac05a69eecbfa9b6e4fe079444dbf7db.

[24] Okin GS, Roberts DA, Murray B, Okin WJ (2001) Practical limits on hyperspectral vegetation discrimination in arid and semiarid environments. Remote Sensing of Environment 77: 212–225.

[25] Richardson JJ, Moskal LM, Kim S-H (2009) Modeling approaches to estimate effective leaf area index from aerial discrete-return LIDAR. Agricultural and Forest Meteorology 149: 1152–1160.

[26] Diggle PJ, Ribeiro PJ (2002) Bayesian Inference in Gaussian Model-based Geostatistics. Geographical and Environmental Modelling 6: 129–146.

[27] Berg TM, Edmunds WE, Geyer AR, et al (1980) Geologic Map of Pennsylvania. 4th Ser., Map 1 Harrisburg, PA: Pennsylvania Geological Survey

[28] Lin H (2006) Temporal stability of soil moisture spatial pattern and subsurface preferential flow pathways in the Shale Hills Catchment. Vadose Zone Journal 5: 317.

[29] Zhu Q, Lin H, Doolittle J (2010) Repeated Electromagnetic Induction Surveys for Determining Subsurface Hydrologic Dynamics in an Agricultural Landscape. Soil Science Society of America Journal 74: 1750.

[30] Takagi K, Lin HS (2011) Temporal Dynamics of Soil Moisture Spatial Variability in the Shale Hills Critical Zone Observatory. Vadose Zone Journal 10: 832–842.

[31] Guo Q, Li W, Yu H, Alvarez O (2010) Effects of topographic variability and lidar sampling density on several DEM interpolation methods. Photogrammetric Engineering and Remote Sensing 76: 701–712.

[32] Tarboton DG (1997) A new method for the determination of flow directions and upslope areas in grid digital elevation models. Water Resources Research 33: 309–319.

[33] Diggle PJ, Moyeed RA, Tawn JA (1998) Model-based Geostatistics. Applied Statistics 47: 299–350.

[34] Ribeiro Jr PJ, Diggle PJ (2001) geoR: A package for geostatistical analysis. R News 1: 14–18.

[35] R Development Core Team (2010) R: A language and environment for statistical computing. Vienna, Austria: R Foundation for Statistical Computing Available: http://www.R-project.org.

[36] Nemani R, Pierce L, Running S, Band L (1993) Forest ecosystem processes at the watershed scale: sensitivity to remotely-sensed leaf area index estimates. International Journal of Remote Sensing 14: 2519–2534.

[37] Tissue DT, Wright SJ (1995) Effect of Seasonal Water Availability on Phenology and the Annual Shoot Carbohydrate Cycle of Tropical Forest Shrubs. Functional Ecology 9: 518–527.

[38] Kikuzawa K (1983) Leaf survival of woody plants in deciduous broad-leaved forests. 1. Tall trees. Canadian Journal of Botany 61: 2133–2139.

[39] Kikuzawa K (1988) Leaf Survivals of Tree Species in Deciduous Broad-Leaved Forests. Plant Species Biology 3: 67–76.

[40] Seiwa K (1999) Changes in Leaf Phenology are Dependent on Tree Height in Acer mono, a Deciduous Broad-leaved Tree. Annals of Botany 83: 355–361.

[41] Sigurdsson B (2001) Elevated [CO2] and nutrient status modified leaf phenology and growth rhythm of young Populus trichocarpa trees in a 3-year field study. Trees - Structure and Function 15: 403–413.

[42] Jin M, Zhang DL (2002) Observed variations of leaf area index and its relationship with surface temperatures during warm seasons. Meteorology and Atmospheric Physics 80: 117–129.

[43] Estrella N, Sparks TH, Menzel A (2009) Effects of temperature, phase type and timing, location, and human density on plant phenological responses in Europe. Clim Res 39: 235–248.

[44] Vitasse Y, Delzon S, Dufrêne E, Pontailler JY, Louvet JM, et al (2009) Leaf phenology sensitivity to temperature in European trees: Do within-species populations exhibit similar responses? Agricultural and Forest Meteorology 149: 735–744.

[45] Reich PB, Uhl C, Walters MB, Prugh L, Ellsworth DS (2004) Leaf demography and phenology in Amazonian rain forest: a census of 40 000 leaves of 23 tree species. Ecological Monographs 74: 3–23.

[46] Vivoni ER (2012) Diagnosing Seasonal Vegetation Impacts on Evapotranspiration and Its Partitioning at the Catchment Scale during SMEX04–NAME. Journal of Hydrometeorology 13: 1631–1638.

[47] White MA, Thornton PE, Running SW (1997) A continental phenology model for monitoring vegetation responses to interannual climatic variability. Global Biogeochemical Cycles 11: 217–234.

[48] Williams RJ, Myers BA, Muller WJ, Duff GA, Eamus D (1997) Leaf Phenology of Woody Species in a North Australian Tropical Savanna. Ecology 78: 2542–2558.

[49] Borchert R (1994) Soil and stem water storage determine phenology and distribution of tropical dry forest trees. Ecology 1437–1449.

[50] Borchert R (1994) Induction of rehydration and bud break by irrigation or rain in decidous trees of a tropical dry forest in Costa Rica. Trees-Structure and Function 8: 198–204.

[51] Takagi K, Lin HS (2012) Changing controls of soil moisture spatial organization in the Shale Hills Catchment. Geoderma 173–174: 289–302.

