# Supplementary figures and images for: Spatial Distribution of Tree Species Governs the Spatio-Temporal Interaction of Leaf Area Index and Soil Moisture across a Forested Landscape
Source: PLoS One. 2013 Mar 12;8(3):e58704. doi: 10.1371/journal.pone.0058704 (PMC3595292; doi:10.1371/journal.pone.0058704)

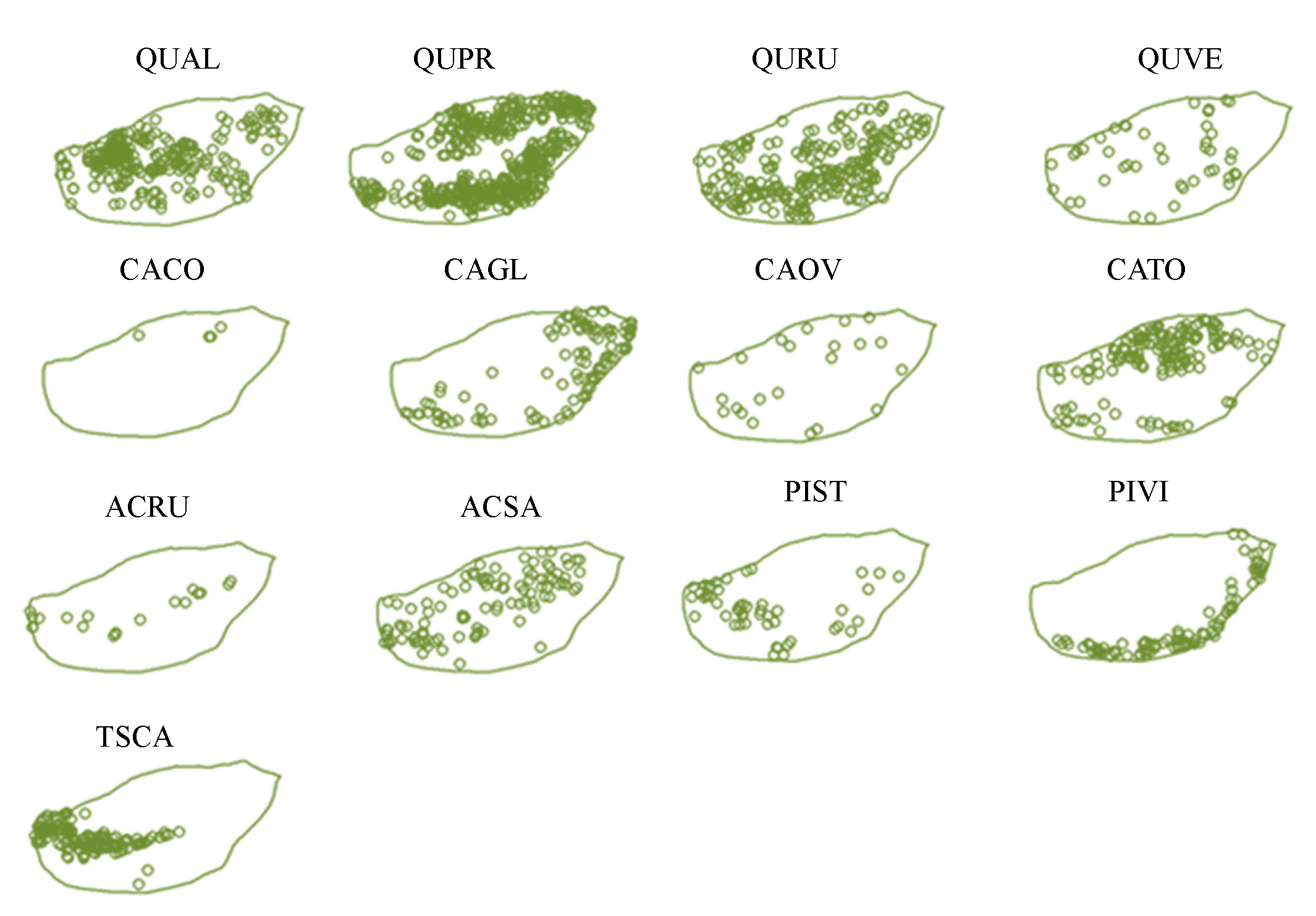

Supplement: Figure S1 — Spatial distribution of deciduous (oaks [ Quercus alba- QUAL, Q. prinus- QUPR, Q. rubra- QURU, Q. velatina -QUVE], hickories [ Carya cordiformis -CACO, C. glabra- CAGL, C. ovata -CAOV, C. tomentosa CATO], maples [ Acer saccharum- ACSA, A. rubrum -ACRU]) and conifer (pines [ Pinus strobus -PIST, P. virginiana -PIVI] and eastern hemlock [ Tsuga canadensis- TSCA]) trees across the Susquehanna Shale Hills Critical Zone Observatory. (TIF) [file pone.0058704.s001.tif]

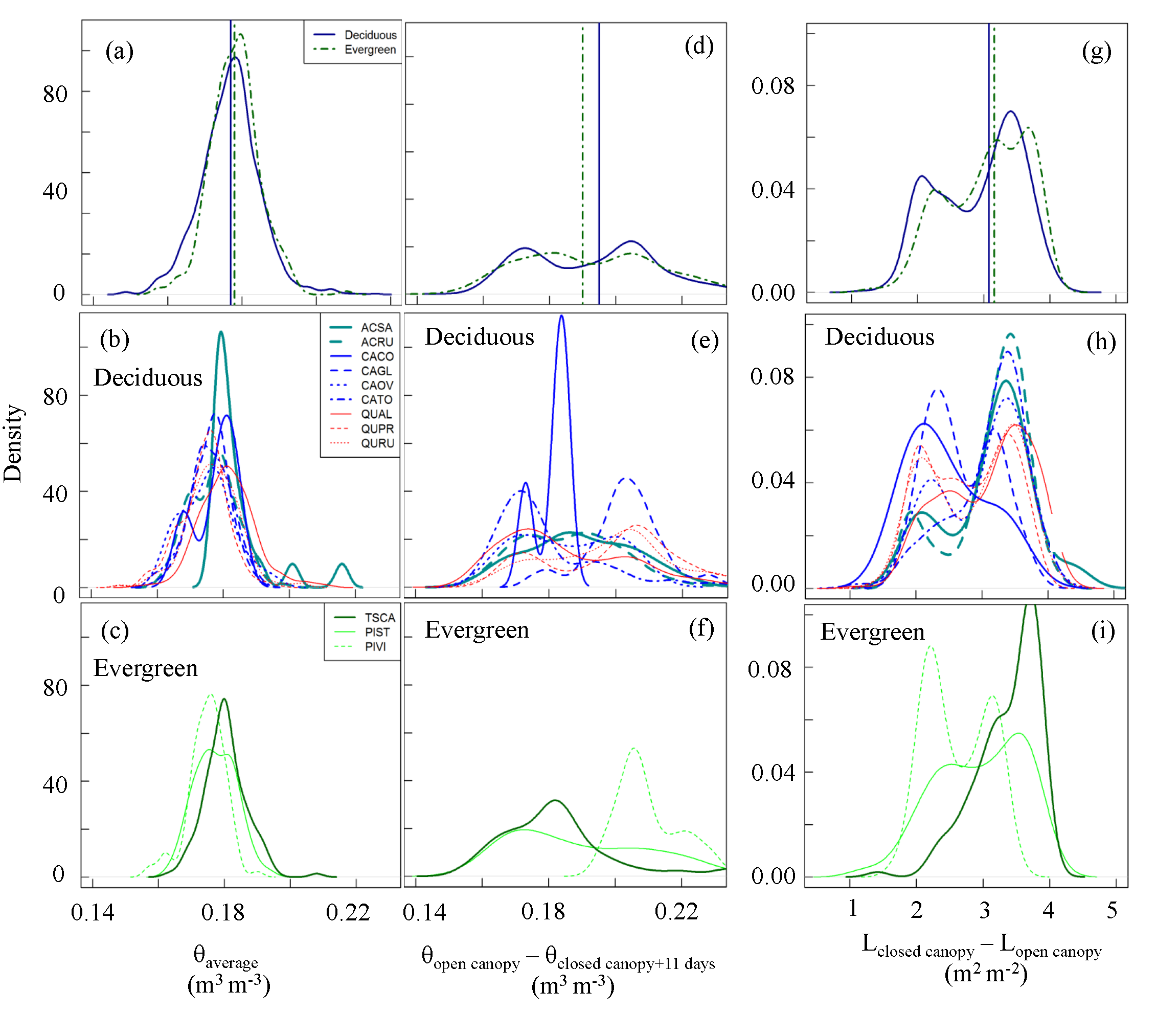

Supplement: Figure S2 — Distribution of deciduous (Acer saccharum-ACSA, A. rubrum-ACRU, Carya cordiformis-CACO, C. glabra-CAGL, C. ovata-CAOV, C. tomentosa-CATO, Quercus alba-QUAL, Q. prinus-QUPR, Q. rubra-QURU) and evergreen (Tsuga canadensis-TSCA, Pinus strobus-PIST, P. virginiana-PIVI) species across a gradient of (a–c) time averaged volumetric soil (10 cm) water content (θaverage: m3 m−3), (d–f) change in θ from budburst to closed canopy, 11 day were added to closed canopy to account for the lag between L and θ; and (g–i) change in leaf area index (L: m2 m−2) from budburst to closed canopy. Vertical lines represent the center (mode) of the distribution. Density curves were calculated from the values sampled at each tree location (total trees = 1832). (TIF) [file pone.0058704.s002.tif]

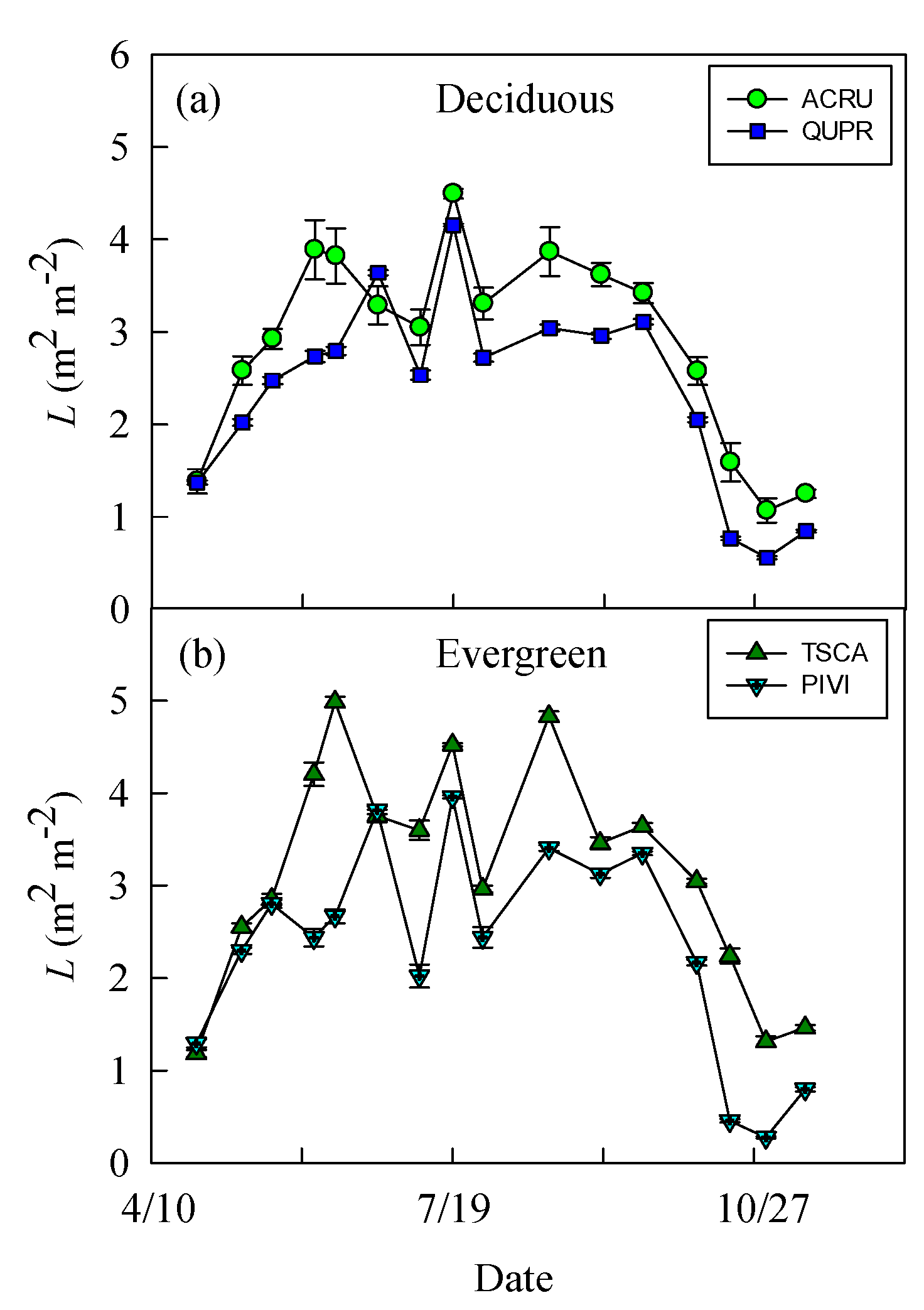

Supplement: Figure S3 — Examples of different timing of budburst, maturity and senescence in (a) deciduous (maple [ Acer rubrum- ACRU] and oak [ Quercus prinus- QUPR], and (b) evergreen (eastern hemlock [ Tsuga canadensis- TSCA] and pine [ Pinus virginiana- PIVI] trees. Each point is an average of posterior mean of leaf area index (L: m2 m−2) for all trees within a species across the landscape and error bar represents standard error of mean. (TIF) [file pone.0058704.s003.tif]

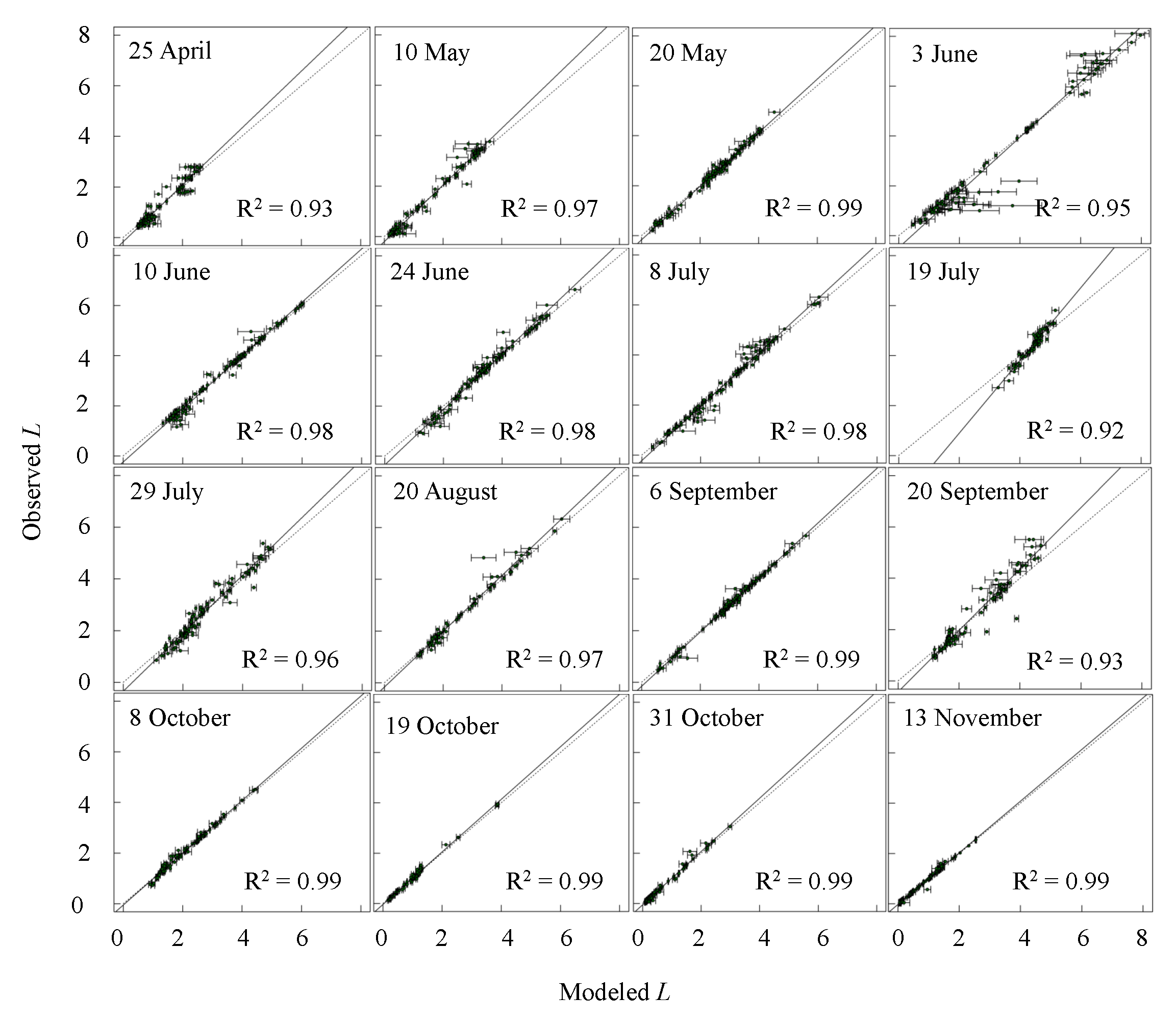

Supplement: Figure S4 — Figure showing leave-one-out cross-validation to assess the model goodness of fit. Dotted line represents 1:1 line and solid line is the slope of linear regression between observed and modeled value of Leaf area index (L: m2 m−2). (TIF) [file pone.0058704.s004.tif]

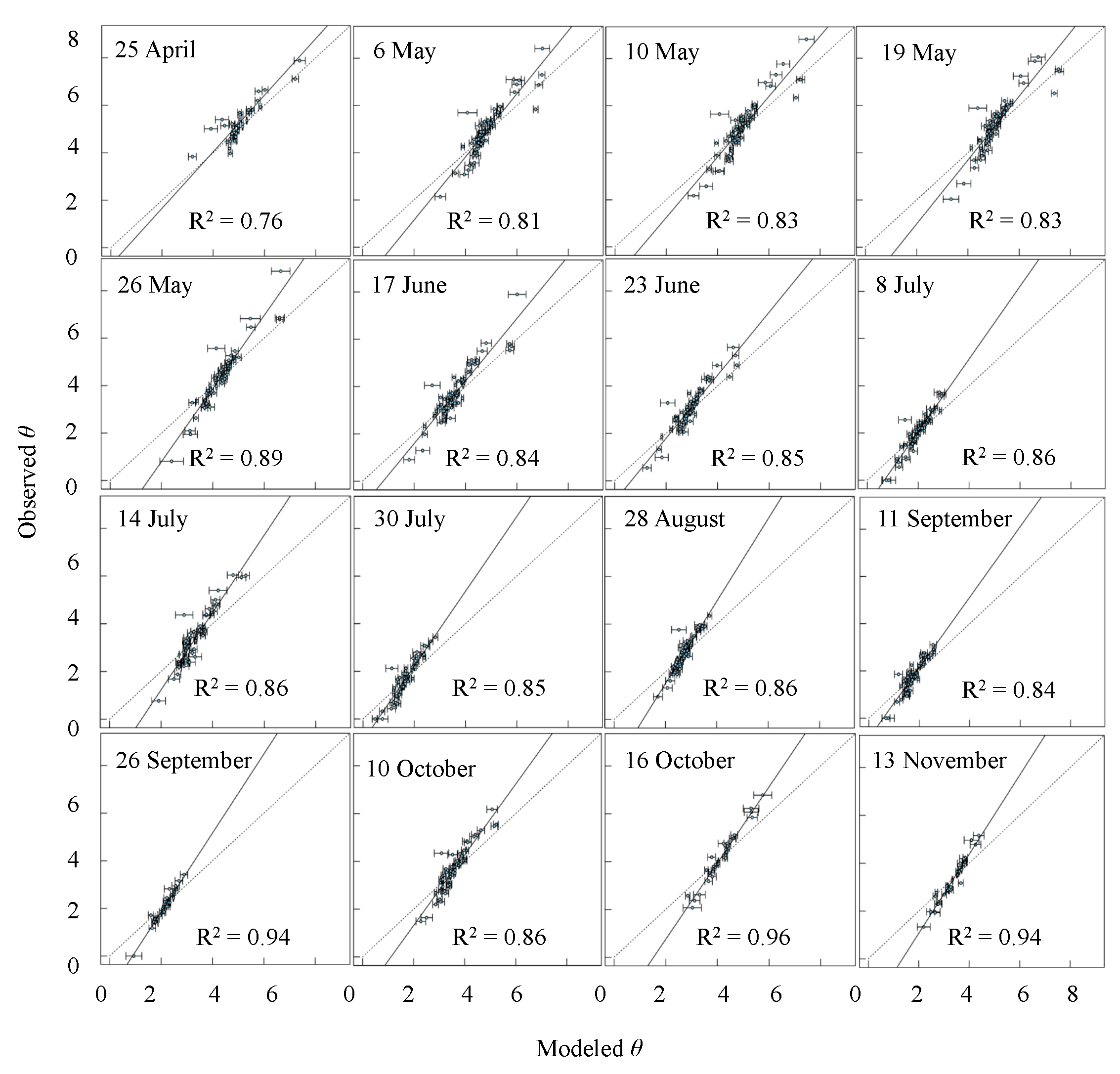

Supplement: Figure S5 — Figure showing leave-one-out cross-validation to assess the model goodness of fit. Dotted line represents 1:1 line and solid line is the slope of linear regression between observed and modeled value of surface (10 cm) soil water content (θ: m3 m−3). (TIF) [file pone.0058704.s005.tif]
